# Venous saturation guided postoperative care for pediatric cardiac surgical patients: the body wants oxygen!

**DOI:** 10.1016/j.bjane.2025.844641

**Published:** 2025-05-16

**Authors:** Rohit S. Loomba

**Affiliations:** Ann & Robert H. Lurie Children’s Hospital, Northwestern University Feinberg School of Medicine, Chicago, Illinois

Dear Editor,

We read the study by Ferreira and colleagues regarding goal-directed therapy using continuous superior caval venous oxygen saturation with great interest.^1^ The authors instituted randomized 65 patients to either venous-saturation guided care or standard care. Those who were in the venous-saturation guided care group had a venous saturation goal of 65% if they had an acyanotic lesion and 55% if they had a cyanotic lesion. They found that lactate clearance (primary endpoint) did not differ between the two groups but that the duration of mechanical ventilation, total use of vasoactive medications, and intensive care unit length of stay were all lower in the venous-saturation guided care group.

These data indicate a few things. Primarily, they highlight that venous-saturation guided care may be associated with improved clinical outcomes. Secondly, it highlights that lactate clearance may not be associated with clinical outcomes at all. These findings are not particularly surprising, although still not widely accepted.

The human body and its constituent organs need oxygen to fuel metabolism and power cellular processes and inadequate systemic oxygen delivery (of which venous saturation is a marker) is associated with dysfunction in nearly any organ system. Low venous saturation (or surrogate near infrared spectroscopy) has been demonstrated to be associated with developmental delay, cardiac dysfunction, extubation failure, feeding intolerance, necrotizing enterocolitis, and hepatic insufficiency. Furthermore, oximetric indices such as venous saturation or near infrared spectroscopy have been demonstrated to have better prognostic ability than blood pressure or serum lactate. Both blood pressure and serum lactate are often used as primary metrics to help guide clinical care after pediatric cardiac surgery while venous saturation still has not been as widely adopted for this purpose. This does not make logical sense as the Fick principle clearly demonstrates that the venous saturation is inversely related to cardiac output.^2^^,^^3^

Blood pressure on the other hand, is the product of cardiac output and systemic vascular resistance, the latter of which cannot be clinically monitored in a clinically meaningful way. Thus, changes in blood pressure are inaccurately assumed to be directly related to cardiac output but without knowing how systemic vascular resistance is changing, simultaneously, this is a misguided assumption. A decrease in cardiac output with an increase in systemic vascular resistance can lead to higher blood pressure and be falsely reassuring. Conversely an increase in cardiac output with a decrease in systemic vascular resistant can lead to lower blood pressure and be falsely alarming.^2^

Serum lactate has been demonstrated to have poor correlation with systemic oxygen delivery in the pediatric cardiac surgical patient. Lactate is produced by several mechanisms for several reasons in the body that go well-beyond simply as a response to anerobic metabolism and, thus, the poor correlation of lactate with oximetric indices shouldn’t be surprisingly.^4^^,^^5^

If oxygen is what the body and its constituent organs need then why utilize indirect markers of systemic oxygen delivery when oximetric indices are widely available. Whether it be venous saturation monitoring by blood gas, central venous catheter optical technology, or near infrared spectroscopy, monitoring the adequacy of systemic oxygen delivery can be done using oximetric indices.

Now Ferreira and colleagues systematically demonstrate that not only is this monitoring possible but that utilizing this to guide care is feasible and associated with benefits. Simply because blood pressure has been measured since the late 1700s should not mean that we shouldn’t begin to shift our reliance on it and to venous saturation guided care in the 2020s. History and groupthink should be placed aside, and objective data should guide the evolution of clinical care. Ferreira and colleagues should be applauded for their efforts to produce meaningful data regarding venous-saturation guided care.

## Conflicts of interest

The authors declare no conflicts of interest.
